# Genome-Wide Identification and Expression Analysis of the MADS-Box Gene Family in Sweet Potato [*Ipomoea batatas* (L.) Lam]

**DOI:** 10.3389/fgene.2021.750137

**Published:** 2021-11-17

**Authors:** Zhengwei Shao, Minhong He, Zhipeng Zeng, Yanzhu Chen, Amoanimaa-Dede Hanna, Hongbo Zhu

**Affiliations:** College of Coastal Agricultural Sciences, Guangdong Ocean University, Zhanjiang, China

**Keywords:** sweet potato, MADS-box, MIKC type, bioinformatics analysis, growth development, expression analysis

## Abstract

MADS-box gene, one of the largest transcription factor families in plants, is a class of transcription factors widely present in eukaryotes. It plays an important role in plant growth and development and participates in the growth and development of flowers and fruits. Sweet potato is the seventh most important food crop in the world. Its tuberous roots, stems, and leaves contain a large number of proteins, lipids, carotenoids, anthocyanins, conjugated phenolic acids, and minerals, which have high edible, forage, and medicinal value, and is also an important energy crop. At present, MADS-box genes in sweet potato have rarely been reported, and there has been no study on the genome-wide identification and classification of MADS-box genes in *Ipomoea batatas*. This study provided the first comprehensive analysis of sweet potato MADS-box genes. We identified 95 MADS-box genes, analyzed the structure and protein of sweet potato MADS-box genes, and categorized them based on phylogenetic analysis with *Arabidopsis* MADS-box proteins. Chromosomal localization indicated an unequal number of MADS-box genes in all 14 chromosomes except LG3, with more than 10 MADS-box genes located on chromosomes LG7, LG11, and LG15. The MADS domain and core motifs of the sweet potato MADS-box genes were identified by motif analysis. We identified 19 MADS-box genes with collinear relationships and analyzed duplication events. *Cis*-acting elements, such as light-responsive, auxin-responsive, drought-inducible, and MeJA-responsive elements, were found in the promoter region of the MADS-box genes in sweet potato, which further indicates the basis of MADS-box gene regulation in response to environmental changes and hormones. RNA-seq suggested that sweet potato MADS-box genes exhibit tissue-specific expression patterns, with 34 genes highly expressed in sweet potato flowers and fruits, and 19 genes highly expressed in the tuberous root, pencil root, or fibrous root. qRT-PCR again validated the expression levels of the 10 genes and found that *IbMADS1*, *IbMADS18*, *IbMADS19*, *IbMADS79*, and *IbMADS90* were highly expressed in the tuberous root or fibrous root, and *IbMADS18*, *IbMADS31*, and *IbMADS83* were highly expressed in the fruit. In this study, the molecular basis of MADS-box genes of sweet potato was analyzed from various angles. The effects of MADS-box genes on the growth and development of sweet potato were investigated, which may provide a certain theoretical basis for molecular breeding of sweet potato.

## 1 Introduction

MADS-box family gene is a kind of transcription factor (TF) widely existing in eukaryotes. MADS is the first letter abbreviation of Mini chromosome maintenance 1 (MCM1) gene in *Saccharomyces cerevisiae* (Passmore et al., 1988), AGAMOUS (AG) gene in *Arabidopsis thaliana* (Yanofsky et al., 1990), DEFICIENS (DEF) gene in *Antirrhinum majus* (Sommer et al., 1990), and serum response factor (SRF) gene in human serum (Norman et al., 1988). It is one of the largest TF families in plants and plays an important role in plant growth and development. Based on phylogenetic analysis, MADS-box genes in plants are categorized into type I and type II (Parenicova et al., 2003). Type I MADS-box gene, also known as M Type, usually has one to two exons, encoding proteins with highly conserved MADS domain. According to the differences in MADS domain, it is further divided into M*α*, M*β*, and M*γ* subfamilies (De Bodt et al., 2003). Type II gene, also known as MIKC type MADS-box gene, is a kind of plant-specific MADS-box gene with six to eight exons. The encoded protein contains four conserved domains: MADS-box (M domain), Intervening domain (I domain), Keratin-like domain (K domain), and C-terminal domain (Nam et al., 2005).

The completion of the whole genome sequences of many plant species has led to the identification and characterization of important gene families. MADS-box genes are integral to the ABCDE model of flowering regulation. Fan et al. identified three flower development class E genes, *AGL2*, *AGL4*, and *AGL9* (Fan et al., 1997). Class E genes cooperate with several other classes to control calyx, petal, stamen, carpel, and ovule development (Theissen, 2001). *AP3* homolog genes in *Chrysanthemum* play a role in the development of ray floret and disc floret in chrysanthemum (Won et al., 2021), and nitrate signaling-associated TFs such as *AGL8*, *AGL21*, and *LBD29* are involved in CmANR1-modulated control of root development. In addition, *CmANR1* also acts as a positive regulator to control shoot growth and development (Sun et al., 2021). Wang et al. found 15 tomato MADS-box genes involved in floral organ identification and five tomato MADS-box genes related to fruit development (Wang et al., 2019). *AGL17* towards the elongation zone is expressed in lateral root cap epidermal cells; *AGL12* and *AGL21* are also expressed in the central cylinder of differentiated roots. Both are expressed in developing embryos of *Arabidopsis* (Burgeff et al., 2002).

Sweet potato [*Ipomoea batatas* (L.) Lam.], 2n = 6*x* = 90, is an annual herb, and its underground parts are round, oval, or spindle-shaped tuberous roots. The shape, skin color, and flesh color of the tuberous root vary with varieties or soil (Wu et al., 1998). Sweet potato is also the seventh important food crop in the world, with high yield and wide use. The tuberous roots, stems, and leaves contain a large number of proteins, lipids, carotenoids, anthocyanin, conjugated phenolic acids, and minerals, which have high edible, feed, and medicinal values, and is also essential energy crop (Wang et al., 2016). In 2002, Kim et al. cloned two MADS-box genes named *IbMADS3* and *IbMADS4* from sweet potato for the first time and found that these two MADS-box genes were significantly expressed in pencil roots and tuberous roots of sweet potato (Kim et al., 2002). In 2005, Kim et al. reported three MADS-box genes of *IbMADS79*, *IbAGL17*, and *IbAGL20*; and they found that *IbMADS79* was only expressed in roots of sweet potato (Kim et al., 2005). Subsequently, Ku et al. (2008) cloned *IbMADS1*, indicating that *IbMADS1* was involved in the initiation of tuberous root differentiation of sweet potato. Dong et al. (2018) cloned nine MADS-box TFs in 2018 and found that *IbMBP2*, *IbMBP3*, *IbMBP4*, and *IbMBP9* showed high expression during storage roots development and were upregulated by storage roots development-related hormones. In 2020, Zhu et al. identified 37 MADS-box genes in *Ipomoea trifida*, the close wild ancestor of sweet potato. They found that MIKCC genes may be significant for regulating the floral organ development in *I. trifida* (Zhu et al., 2020). The MADS-box genes within the sweet potato genome have not been completely mined; the function and mechanism of action need to be verified; and the identification of more MADS-box genes is beneficial to elucidating the molecular mechanism of growth and development of sweet potato, providing a certain theoretical basis for molecular breeding.

## 2 Materials and Methods

### 2.1 Identification and Gene Structure Analysis of MADS-Box Family Members in Sweet Potato

The sweet potato genomic information was derived from *I. batatas* “Taizhong 6” genomic data provided by the Ipomoea genome hub (https://ipomoea-genome.org/). The MADS-box family HMM model files SRF-TF (PF00319) and K-box (PF01486) were downloaded from the Pfam database (https://pfam.xfam.org/). The whole genome MADS-box family genes of sweet potato were identified by HMMER software (Krejci et al., 2016), and the MADS-box genes were identified by the SMART website (http://smart.embl-heidel-berg.de/) again. The genes containing the MADS-box domain were named from *IbMADS1* to *IbMADS95*, and the three genes matching the published sequences of *IbMADS1* (Ku et al., 2008), *IbMADS4* (Kim et al., 2002), and *IbMADS79* (Kim et al., 2005) were still named as *IbMADS1*, *IbMADS4*, and *IbMADS79*, respectively. *IbMBP1*, *IbMBP4*, and *IbMBP8* (Dong et al., 2018) were named as *IbMADS47*, *IbMADS38*, and *IbMADS51*, respectively, in this study. According to the sweet potato genome gff3 annotation information, GSDS online website was used (http://gsds.gao-lab.org/). The MADS-box gene structure of sweet potato was analyzed visually. TBtools (Chen et al., 2020) was used to plot the innovative prediction results visually. TBtools (Chen et al., 2020) was used for structure visualization. According to the annotation information of the sweet potato genome, the MADS-box genes structure of sweet potato was visualized using GSDS online website tool (http://gsds.gao-lab.org/).

### 2.2 MADS-Box Phylogenetic Tree Construction

The *Arabidopsis* MADS-box protein sequence was obtained from the Plant TFDB website (http://planttfdb.gao-lab.org/index.php) (Jin et al., 2017). *I. trifida* MADS-box proteins were obtained according to the research of Zhu et al. (Zhu et al., 2020). The MADS-box genes of sweet potato were divided into two categories, type I and type II, based on the phylogenetic analysis of the MADS gene with *Arabidopsis*. ClustalW was used to compare the two types of MADS-box proteins; a neighbor-joining (NJ) tree was constructed using bootstrap analysis with 1,000 replicates, the amino acid p-distance model, missing data treatment option set at partial deletion, and a site coverage cutoff of 60%; and MEGA-X (Kumar et al., 2018) was used to construct the NJ phylogenetic tree (Saitou and Nei, 1987). The parameters for constructing the evolutionary tree were as follows: Poisson model, uniform rates, Sam (homogeneous), and pairwise deletion. According to the classification method of Sheng et al. (2019), the MADS-box gene subfamily of sweet potato was categorized based on the MADS-box gene subfamily of *Arabidopsis*, using Evolview online tools (https://www.evolgenius.info/evolview/) to beautify the tree.

### 2.3 Chromosomal Localization of MADS-Box Genes

According to the annotation information of the sweet potato genome downloaded from the Ipomoea genome hub, the positions of 95 sweet potato MADS-box genes on chromosomes were obtained, and the chromosome mapping was performed using Map Chart (Voorrips, 2002) software. Origin 8.5 (https://www.originlab.com) was used to draw the distribution cake map of sweet potato MADS-box genes.

### 2.4 Conserved Motif Analysis of MADS-Box Proteins

The sweet potato MADS-box protein sequence was submitted to MEME online tool (http://meme-suite.org/) (Bailey et al., 2009) for the motif prediction. The discovery number of Motif was set to be 8, the width of motif was 6–50, and other default settings were used. The MADS-box motif domain of sweet potato was visualized by TBtools.

### 2.5 *Cis*-Acting Elements Analysis of MADS-Box Genes

According to the annotation information of the sweet potato genome and sweet potato genome sequence, referring to Sumiya (2021) and Zhang et al. (2021), the 2-kb upstream sequence of sweet potato MADS-box genes was submitted to PlantCare website (http://bioinformatics.psb.ugent.be/webtools/plantcare/html/) (Lescot et al., 2002) for *cis*-acting element prediction. The PlantCare analysis results were simplified, and TBtools was used for visualization.

### 2.6 Collinearity Analysis of MADS-Box Genes

According to the annotation information and the whole genome protein sequence of sweet potato, MCScanX (Wang et al., 2012) software was used to analyze the MADS-box collinearity of sweet potato, and Circos (Krzywinski et al., 2009) software was used for visualization.

### 2.7 Expression Analysis of MADS-Box Genes

The tuberous root (greater than 10 mm in diameter), pencil root (2–5 mm in diameter), fibrous root (diameter of less than 1.5 mm), flower (an incompletely opened bud), fruit (the peel is green and the fruit form full), and stem tip (1–2 rachises of the rolled leaf at the stem apex, excluding the rachis where the expanded leaf is located) of “Jishu 26” (*I. batatas* cv. Ji 26) cultivated in an open field for 100 days were selected. After rapid freezing in liquid nitrogen, with dry ice sent to Biomarker Technologies Company (Beijing, China, https://www.biocloud.net) for total RNA extraction, library construction, and transcriptome sequencing, raw data were uploaded to National Center for Biotechnology Information (NCBI) project PRJNA744414; the expression of sweet potato MADS-box genes was analyzed based on genome information and local blast; the FPKM value (Florea et al., 2013) was calculated to reflect the gene expression; and the calculation formula is as follows: FPKM = {cDNA Fragments\over{Mapped Fragments(Millions) * TranscriptLength(kb)}}. Pheatmap package and ggplot2 package (Ito and Murphy, 2013) of R Statistics (https://www.r-project.org) were used for data processing and visualization.

### 2.8 Verification of Gene Expression by qRT-PCR

The tuberous root is the main harvest organ of sweet potato. According to the expression profile of the MADS-box gene in different parts, several genes highly expressed in tuberous root were selected for qRT-PCR expression verification. The double-stranded cDNA of fibrous root (F.R, sampling details were the same as 1.7), tuberous root (T.R, sampling details were the same at 1.7), leaf (L, expanded leaf 1–2 at the apex of stem), stem (S, 1–2 rachises of the expanded leaf at the stem apex, excluding the rachis where the rolled leaf is located), stem tip (S.T, sampling details were the same at 1.7), fruit (F.T, sampling details were the same at 1.7), and other tissues cultivated in open field for 100 days “Jishu 26” were used as templates; *β-actin* was used as a reference gene, using the CFX96™ quantitative PCR instrument to perform the fluorescent quantitative PCR. The reaction program was as follows: predenaturation at 95°C for 3 min, 95°C for 5 s, 60°C for 30 s, 40 cycles; and 95°C for 15 s, 60°C for 6 s, 95°C for 15 s. The reaction system was as follows: 2× universal SYBR green fast qPCR mix (ABclonal Technology, Wuhan, China) for 5 μl, ddH_2_O for 3 μl, cDNA for 1 μl, forward primer for 0.5 μl, and reverse primer for 0.5 μl. Primers were designed using Primer premier 6 (Singh et al., 1998) based on the sequence of the amplified CDs of the MADS-box genes, and the primer sequences are listed in Supplementary Table S1. Three biological replicates and three technical replicates were set up, and relative gene expression was calculated by the 2^−ΔΔCt^ method (Livak and Schmitten, 2001). Data processing and analysis were performed using SPSS software, and column graphs were drawn using origin 8.5.

## 3 Results and Analysis

### 3.1 Identification and Structural Analysis of the MADS-Box Family of Sweet Potato

**FIGURE 1:**
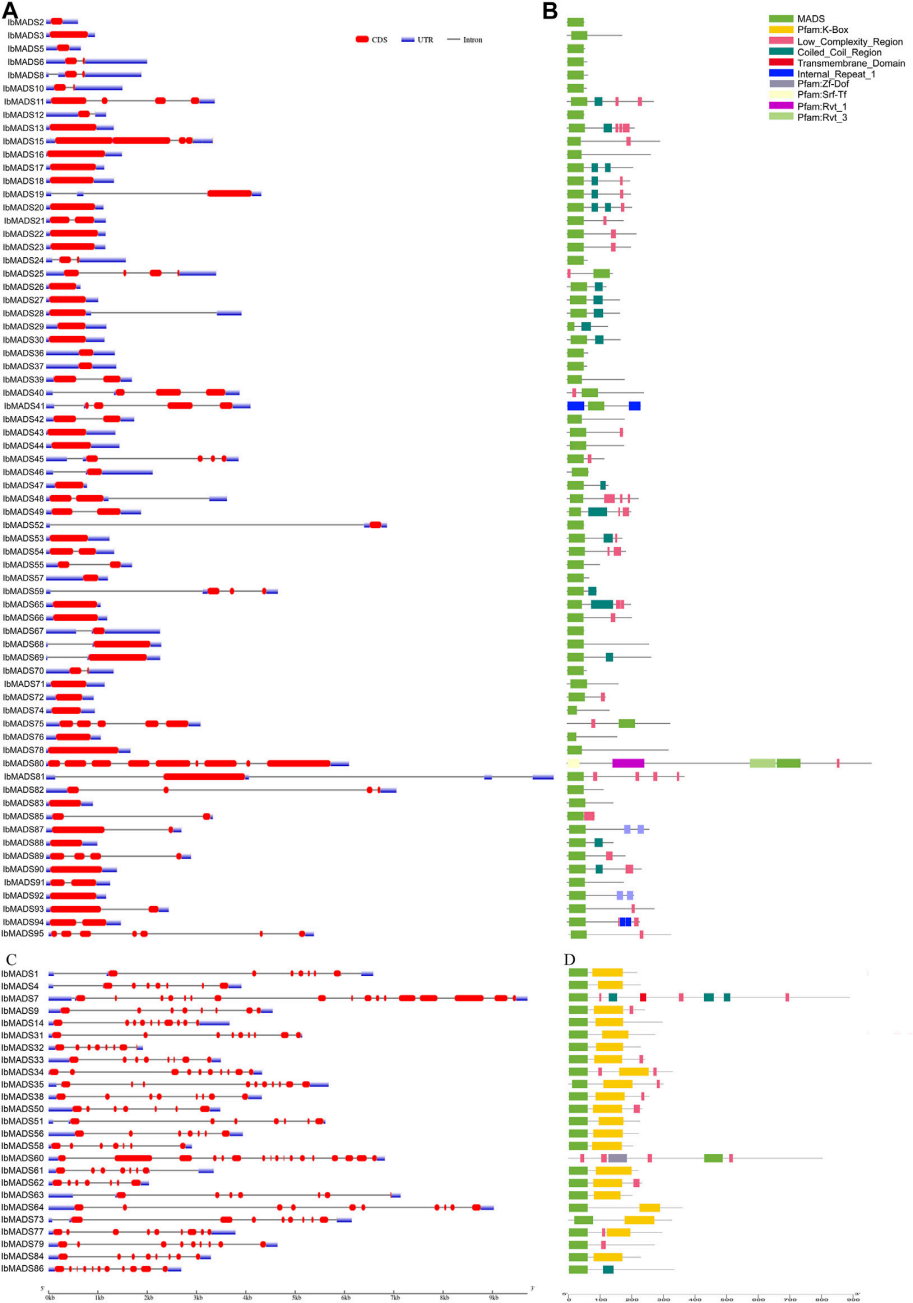
SMART identification and structure of sweet potato MADS-box gene. **(A)** Type I gene structure. **(B)** Type I protein structure prediction by SMART. **(C)** Type II gene structure. **(D)** Type II protein structure prediction by SMART.

A total of 95 MADS-box genes were identified from the whole genome of sweet potato and named according to *IbMADS1*–*IbMADS95* (Supplementary Table S2). According to the gene structure, the sweet potato MADS-box genes were divided into two categories: type I and type II (Figure 1). The different types of MADS-box gene structures of sweet potato have apparent differentiation. Most type I genes are less than 2 kb in length and have one to two long fragment exons. On the other hand, the type II gene is usually over 3 kb and contains more than six exons, and most exonic fragments are short. Smart protein structure prediction found that the sweet potato MADS-box gene contains other domains in addition to the MADS domain. Most type I genes contained variable numbers of low-complexity region (LCR) structures and coiled-coil region (CCR) structures. The 21 type II genes contained a K-box domain; the K-box domain was commonly found to be associated with SRF-type TFs, also the signature domain of type II MADS-box genes. And the type II genes without a K-box domain usually belong to the MIKC* subfamily.

### 3.2 Phylogenetic Analysis of MADS Proteins

**FIGURE 2:**
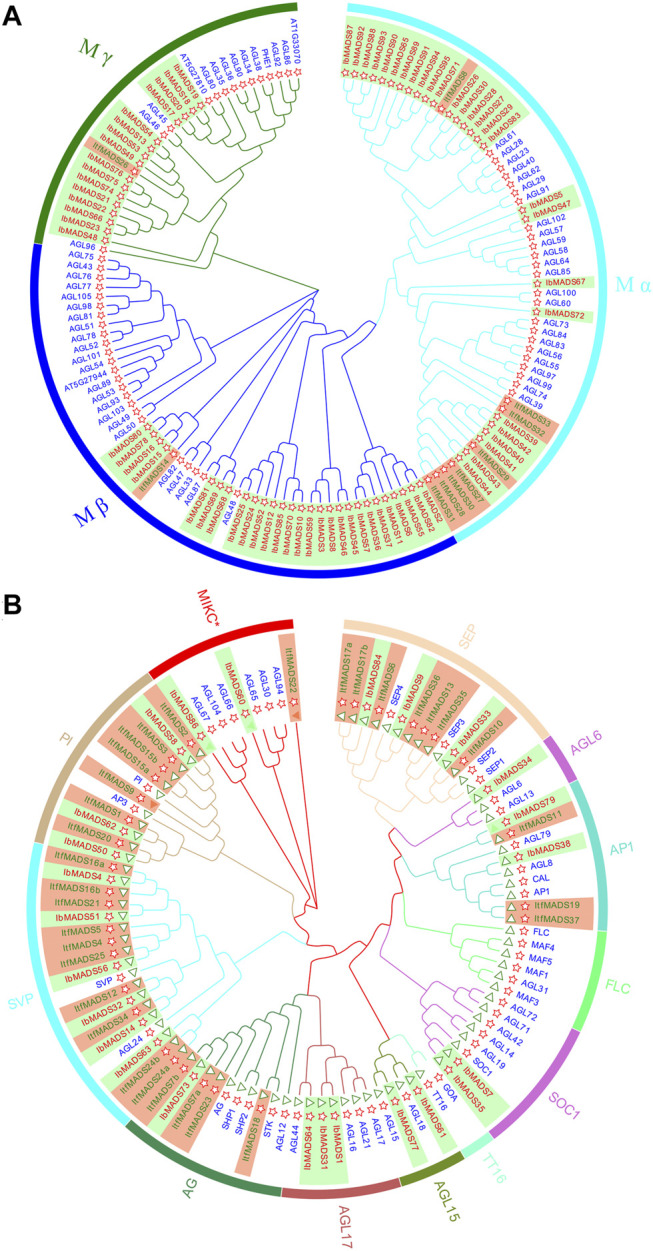
Phylogenetic tree of type I **(A)** and type II **(B)** MADS-box proteins in *Ipomoea batatas*, *Ipomoea trifida*, and *Arabidopsis*. Red ID indicates *I. batatas* gene, green ID indicates *I. trifida* gene, and blue ID indicates *Arabidopsis* gene. The red star indicates the sequence containing the SRF-box domain, and the green triangle indicates the sequence containing K-Box domain.

The NJ phylogenetic tree of MADS-box proteins from *I. batatas*, *I. trifida*, and *Arabidopsis* were constructed by MEGA-X. Based on the subfamily classification of *Arabidopsis* MADS proteins, sweet potato type I MADS proteins were divided into three subfamilies: M*α*, M*β*, and M*γ* (Figure 2A). In comparison, type II MADS proteins were divided into 12 subfamilies (Figure 2B). The type I MADS proteins of sweet potato and *Arabidopsis* were cross-distributed in each subfamily. Among them, both M*α* subfamily and M*β* subfamily had 27 proteins each, and M*γ* subfamily was less abundant with 16 proteins, which was similar to the distribution of the *Arabidopsis* protein subfamily. However, the type I proteins of *I. trifida* were mainly concentrated in M*α* subfamily, with only one gene each in the M*β* and M*γ* subfamilies.

The type II gene of *I. batatas* is phylogenetically closer to that of *I. trifida*, as indicated by gene number and subfamily distribution. The type II gene of *I. batatas* did not appear in the FLC subfamily or TT16 subfamily, and other subfamilies were distributed, with one to six proteins varying in number. The proteins for *I. trifida* are mainly distributed in subfamilies such as SEP, PI, and SVP and show deletions in several subfamilies. Twenty-two type II proteins contain K-box region, while three sweet potato type II proteins have no K-box region, one of them belongs to the AP1 subfamily, and the other two belong to the MIKC* subfamily.

### 3.3 Chromosomal Localization of MADS-Box Genes

**FIGURE 3:**
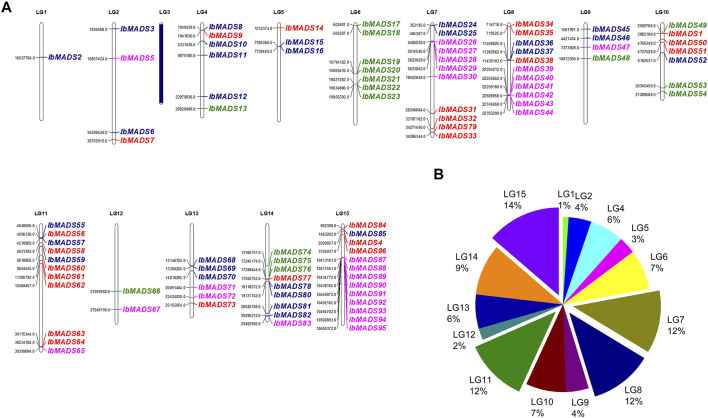
Chromosome location **(A)** and distribution **(B)** of MADS-box gene in sweet potato. The purple name represents M*α* subfamily, the blue name represents M*β* subfamily, the green name represents M*γ* subfamily, and the red name represents type II MADS-box genes. LG3 has no MADS-box gene distribution.

Chromosome localization of 95 MADS-box genes in sweet potato was performed (Figure 3). It was found that except LG3 chromosome, the distribution of MADS-box genes in sweet potato varied in quantity in the other 14 chromosomes. More than 10 MADS-box genes were distributed on LG7, LG8, LG11, and LG15 chromosomes. LG1, LG5, and LG12 chromosomes contain fewer MADS-box genes, only one to three. M*α* subfamily is mainly distributed on chromosomes LG7, LG8, and LG15, with more than five genes. In contrast, the M*β* subfamily (blue) is more fragmented, with one to four genes distributed in all chromosomes except LG16 and LG12. Seven MADS-box genes on LG6 were M*γ* subfamily, and the rest were sporadically distributed on LG4, LG9, LG10, LG12, and LG14. The MIKC subfamily genes are widely distributed, except for LG1, LG3, LG6, LG9, and LG12, which are more numerous on chromosome LG11, with seven genes belonging to the MIKC subfamily. Gene cluster exists for LG6, LG7, LG8, LG14, and LG15, especially in the interval 10413181 to 10505372 of LG15 containing nine MADS-box genes. Gene clusters were present on chromosomes LG6, LG7, LG8, LG14, and LG15. In particular, the interval 10413181 to 10505372 of LG15 contains nine MADS-box gene presence distributed centrally.

### 3.4 Analysis of MADS Proteins Conserve Motif

**FIGURE 4:**
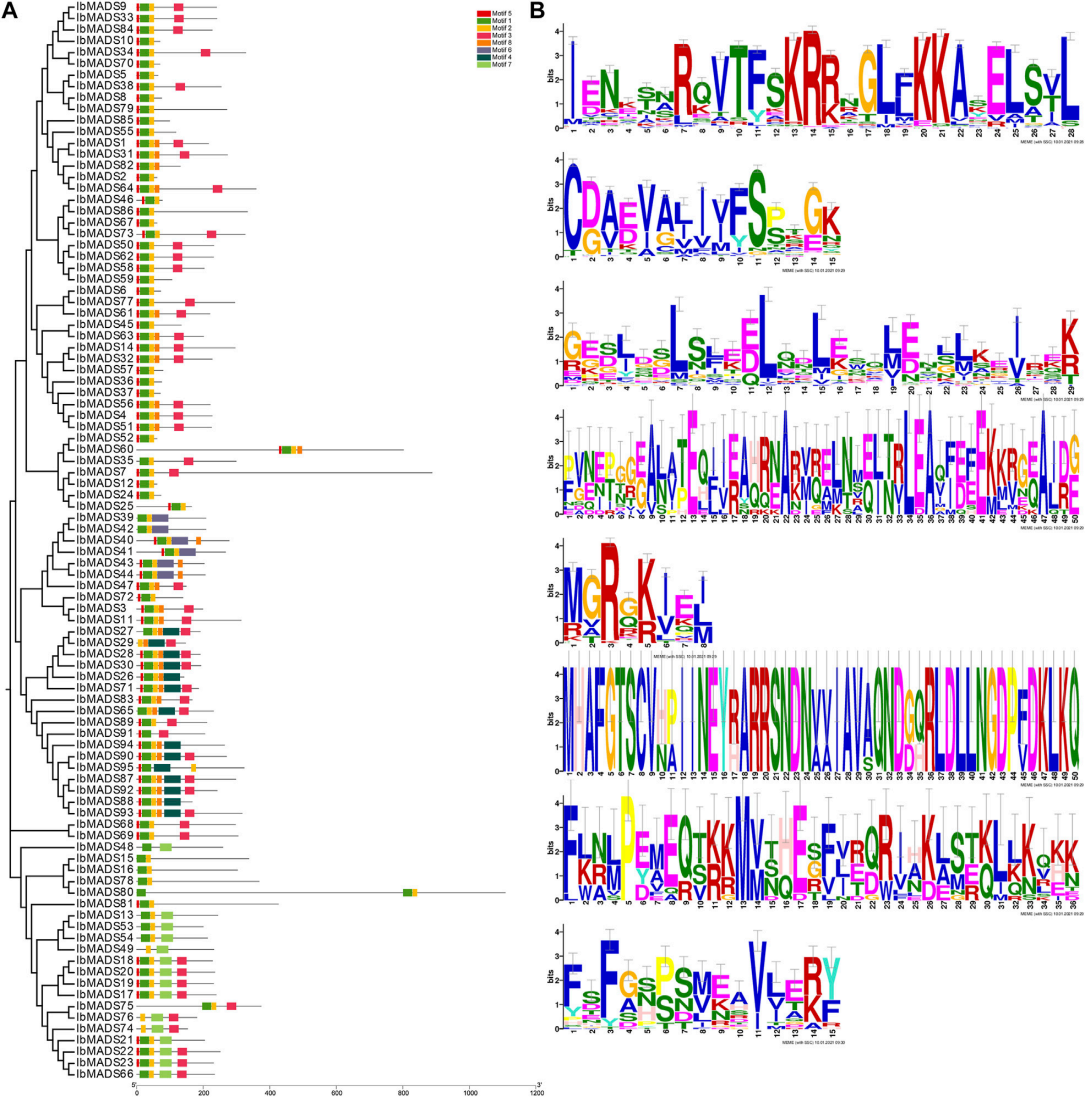
Analysis of MADS-protein motif domain in sweet potato **(A)** and motifs **(B)**.

Conserve motif analysis of sweet potato MADS-protein sequence was performed by online MEME tool. There are some differences in the number of MADS-protein motif in sweet potato. Each protein contains two to six motifs (Figure 4). All genes have motif 1 domain encoding 28 amino acids, and more than 90 genes contain motif 2 and motif 5. The 51 amino acids encoded by these three motifs are the core motifs of MADS-box genes, which are related to the SRF-box region. In addition, the motif 3 domain also plays an important role, and more than 50 proteins contain highly conserved motif 3 domains encoding 29 amino acids, including 22 type II MADS proteins.

### 3.5 Analysis of *Cis*-Acting Elements of MADS-Box Gene Promoter Regions

**FIGURE 5:**
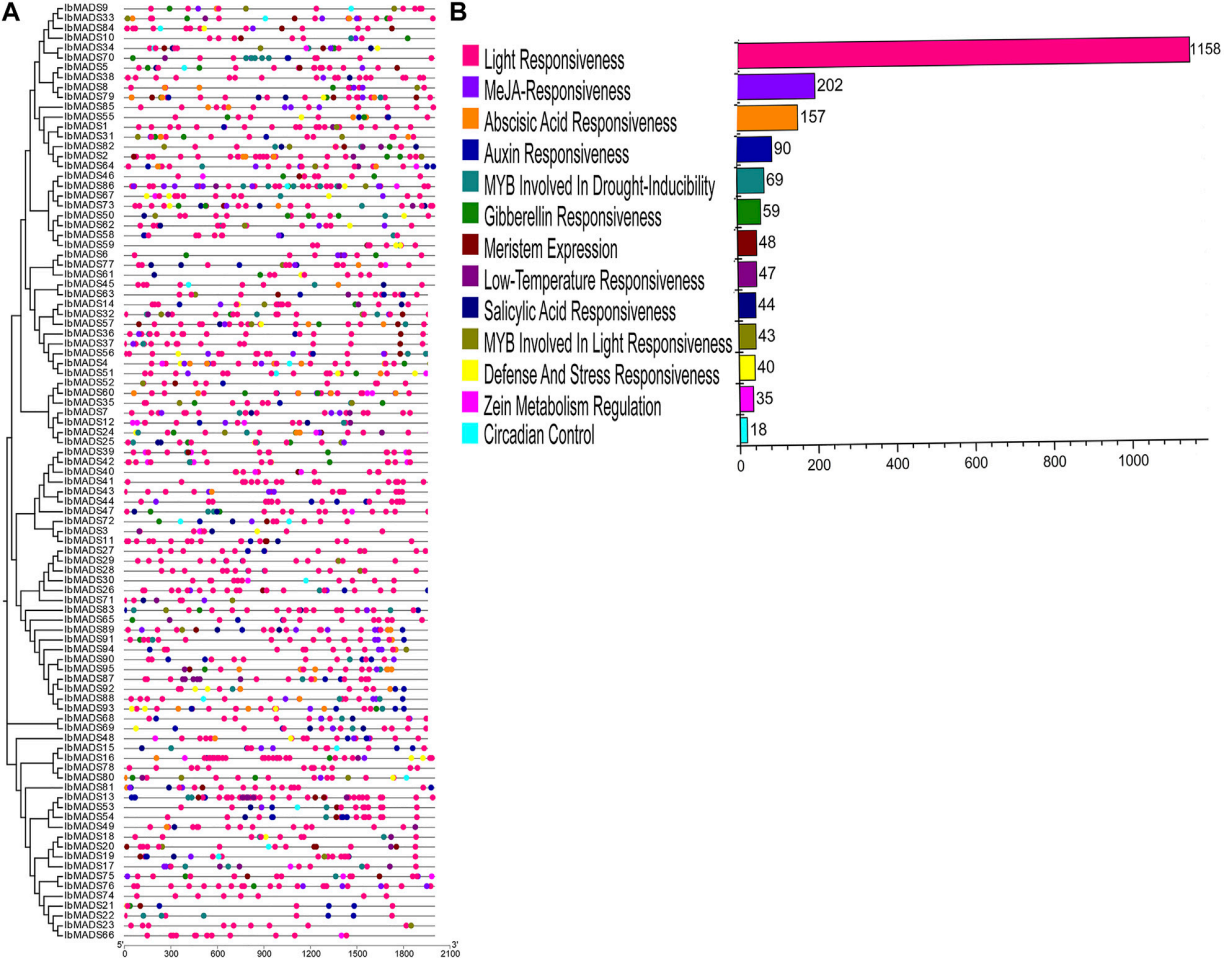
*Cis*-acting elements analysis of sweet potato MADS-box. **(A)** *Cis*-acting element distribution. **(B)** *Cis*-acting element numbers.

The 2-kb upstream CDS sequences were extracted from the promoter regions of 95 MADS-box genes of sweet potato. The *cis*-acting elements in the promoter region of sweet potato MADS-box were predicted by PlantCARE online tool. All sweet potato MADS-box genes contain three to 12 light-responsive elements, including Box 4, G-box, GT1-motif, and TCT-motif. In addition, the promoter regions of sweet potato MADS-box genes are mainly more than 200 MeJA-responsive elements, including CGTCA-motif and TGACG-motif. And ABA-responsive elements (ABRE), auxin-responsive elements (TGA-element and AuxRR-core), and about 100 MYB response elements are involved in drought inducibility and light-responsiveness. In addition, there are a small number of regulatory elements such as circadian control elements, meristem expression elements, salicylic acid-responsive elements, low-temperature-responsive elements, gibberellin-responsive elements, defense, and stress-responsive elements. They indicated that MADS-box genes could respond to various environmental changes and coordinate the average growth and development of sweet potato (Figure 5).

### 3.6 Collinearity Analysis of MADS-Box Gene in Sweet Potato

**FIGURE 6:**
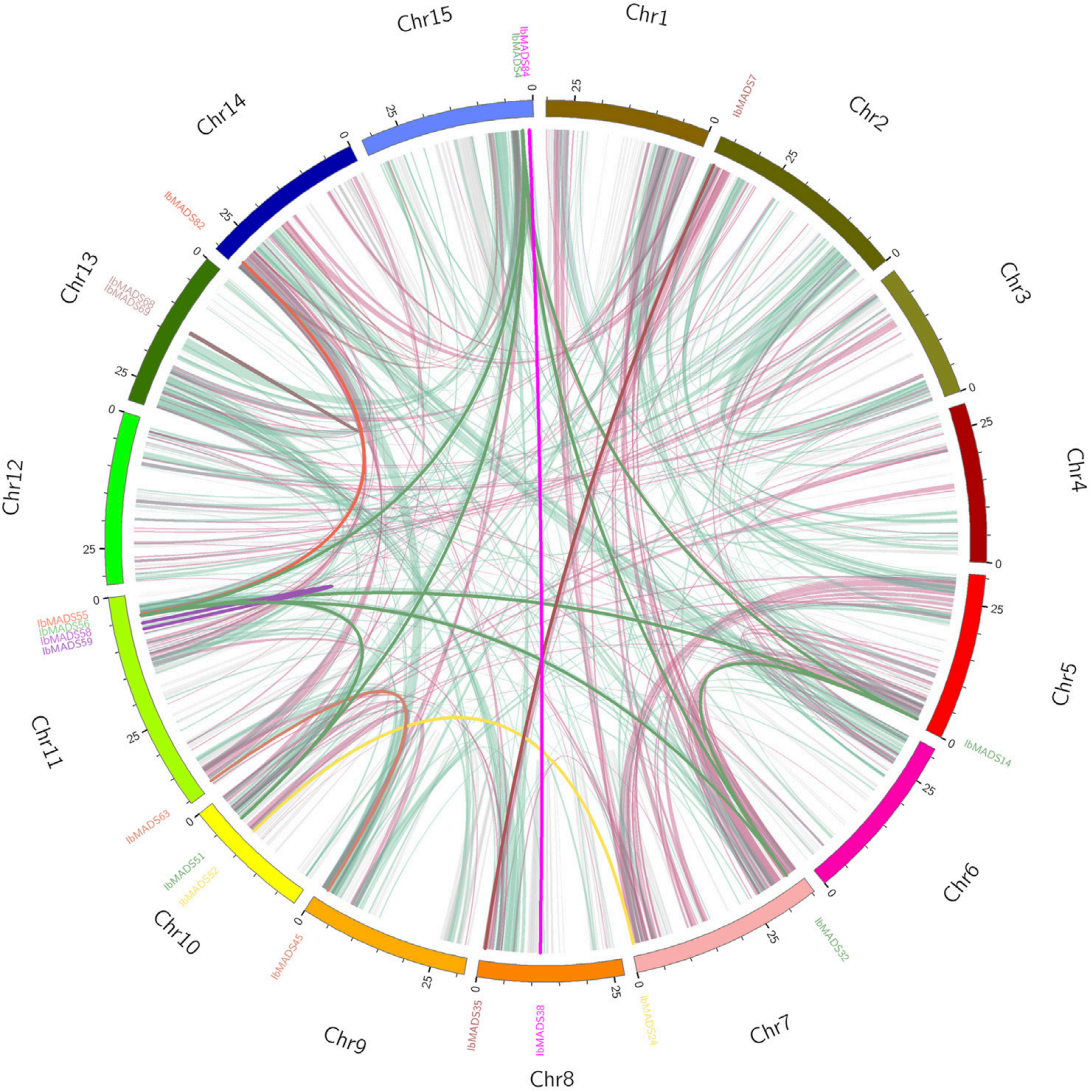
Collinearity analysis of MADS-box gene in sweet potato. The same color name indicates that the gene has a tandem repeat.

Based on the collinearity analysis by MCScanX, a large number of genes were found to be collinear relational (light pink and light green) between and within chromosomes in sweet potato. There are 19 sweet potato MADS-box genes with collinearity, of which 15 genes belonged to inter-chromosome segmental duplications and four genes belonged to tandem duplications. Among them, *IbMADS4*, *IbMADS14*, *IbMADS32*, and *IbMADS56* genes share a reciprocal segmental duplication relationship, with *IbMADS51* showing a segmental duplication relationship with *IbMADS56* and *IbMADS4* but not with *IbMADS14* or *IbMADS32*. There are tandem duplications between *IbMADS58* and *IbMADS59*, and between *IbMADS68* and *IbMADS69*. And there are segmental duplications in the other 10 intergenic pairs. The most MADS-box genes with collinear relationships were on chromosome LG11 with five, and the remaining chromosomes had fewer than two or no MADS-box genes with collinear relationships (Figure 6).

### 3.7 Expression Profiles of MADS-Box Genes in Sweet Potato

**FIGURE 7:**
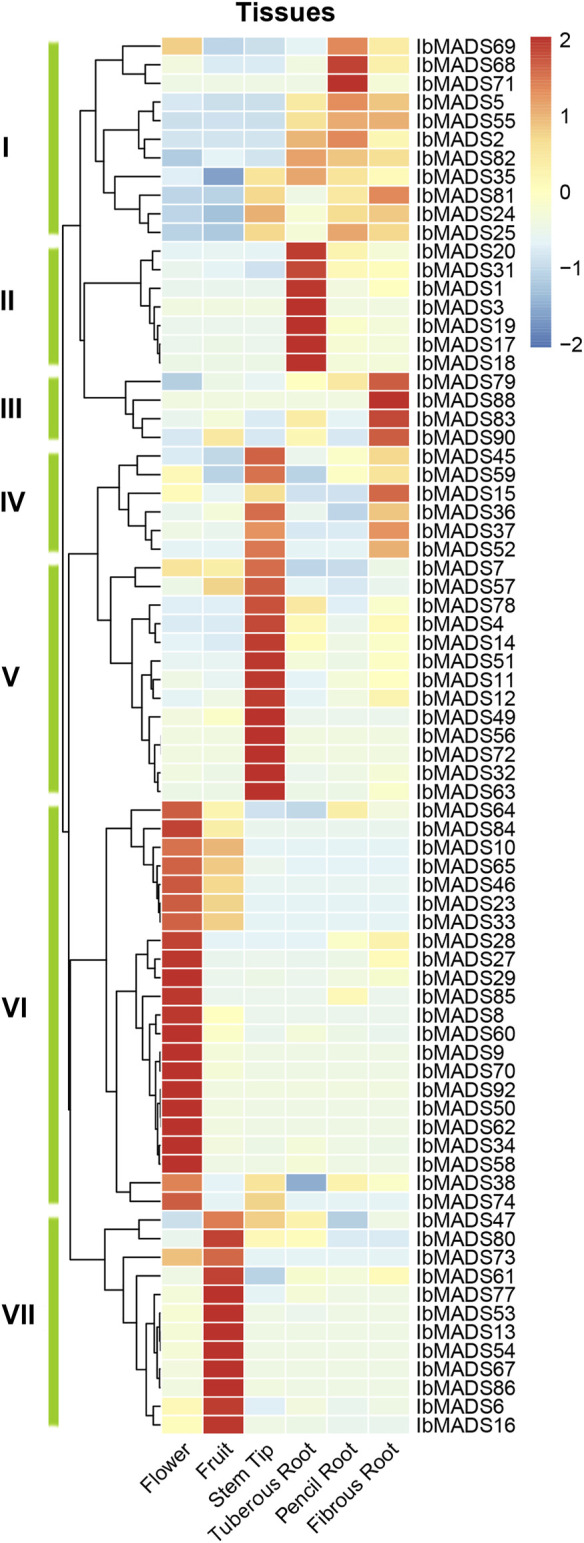
Expression analysis of MADS-box gene in different organisms of sweet potato.

To investigate the expression patterns of MADS-box genes in sweet potato, the 75 MADS-box gene expression profiles were detected in six different tissues by transcriptome sequencing. The results showed that sweet potato MADS-box genes were expressed and differentiated in different tissues (Figure 7). Cluster analysis clustered the sweet potato MADS-box gene expression profiles into seven classes, with class I, II, and III genes mainly expressed in different types of roots. Among them, seven genes in class II were highly and specifically expressed in the tuberous root. The class IV genes were expressed in the stem tip and fibrous root. The class V genes were highly and specifically expressed in the stem tip. The class VI genes were predominantly expressed in flower, and a small proportion of these genes were also somewhat expressed in fruits. The class VII genes are highly and specifically expressed in fruit. Overall, 22 MADS-box genes were highly expressed in flowers, 19 were highly expressed in stem tips, 12 were highly expressed in fruits, and the remaining genes were differentially expressed in different types of roots.

### 3.8 Expression Analysis of MADS-Box Genes by qRT-PCR

**FIGURE 8:**
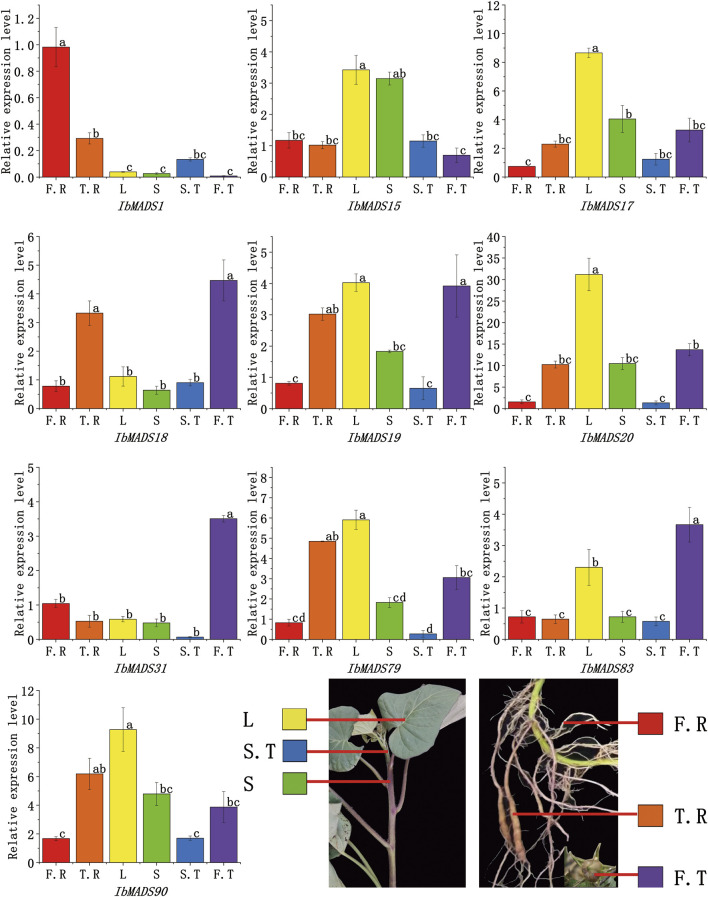
The gene expression levels of some MADS-box genes in different tissues of sweet potato.

qRT-PCR was used to further validate the expression profiles of the 10 genes, which were more highly expressed in roots than other tissues, according to transcriptome sequencing of MADS-box genes in different tissues (Figure 8). Ten genes showed tissue-specific expression at different levels in all tissues; and *IbMADS1*, *IbMADS18*, *IbMADS19*, *IbMADS79*, and *IbMADS90* were highly expressed in the fibrous root or tuberous root. Seven genes were more highly expressed in the leaf, only *IbMADS15* was highly expressed in the stem, and the remaining genes were relatively poorly expressed in the stem. Ten genes were all expressed at lower levels in the stem tip. *IbMADS18*, *IbMADS31*, and *IbMADS83* were significantly expressed in the fruit. Some genes were highly expressed only in a single tissue, and others were not significantly expressed, such as *IbAMDS1*, which was significantly expressed only in the fibrous root; *IbMADS17* and *IbMADS20*, which were highly expressed only in the leaf; and *IbMADS31* and *IbMADS83*, which were significantly expressed only in the fruit. The five remaining genes were more highly expressed in two to three tissues.

## 4 Discussion

MADS-box genes are widely present in eukaryotes. There are 108, 72, 168, and 160 MADS-box genes identified in *Arabidopsis* (Livak and Schmittgen, 2001), *Oryza sativa* (Ray et al., 2007), *Nicotiana tabacum* (Bai et al., 2019), and *Brassica rapa* (Duan et al., 2015). Interestingly, *I. trifida*, a diploid plant belonging to the genus *Ipomoea*, has 37 MADS-box genes (Zhu et al., 2020). In this study, 95 sweet potato MADS-box genes were identified, the number of which was approximately three times that of *I. trifida*. Phylogenetic analysis found that the MADS-box genes of *I. batatas* are not simply tripled by *I. trifida*. We discovered more type I MADS genes in *I. batatas*, and the distribution of type II genes was also more abundant than that of *I. trifida*. Nevertheless, *I. batatas* are missing only in the FLC subfamily and the TT16 subfamily. It is suggested that FLC genes are repressors of flowering which affect multiple pathways for controlling the flowering time (Michaels and Amasino, 1999; Scott et al., 2003). Loss of FLC subfamily genes may indicate that sweet potato vernalization is not regulated by its repression. Consistently, a previous study also showed that the emergence of FLC subfamily members is not detected in *I. trifida* (Zhu et al., 2020). *TT16* encodes a TF involved in the transcriptional regulation of proanthocyanidin biosynthesis in the seed coat (Nesi et al., 2002). The absence of sweet potato TT16 subfamily genes suggests the potential involvement of other pathways for regulating proanthocyanidin biosynthesis in sweet potato.

Moreover, we again found differentiation in the MADS-box gene structure, which may be attributed to the presence of I-domain, K-domain, and C-domain. Type II genes typically exhibit a longer gene length and a greater number of exons, which is also one of the basis for classifying subtypes of MADS-box genes (Kaufmann et al., 2005). Consistent with the structure feature, the type I genes of sweet potato have one to two exons, and most of them range at 1 kb, while the type II genes are generally larger than 3 kb and have more than six exons. In addition, the MADS-box genes of sweet potato are distributed more dispersedly on the chromosome. Their numbers differ from those of chromosomes, which are similar to the chromosome distribution of *Arabidopsis* (Lamesch et al., 2012), *Jatropha curcas* (Tang et al., 2020), *Cardamine hirsuta* (Ghorbani et al., 2020), and *I. trifida*. The MADS-box proteins in sweet potato have a strong motif conservation among various subfamilies, with different numbers and motifs. The core motifs are motif 1, motif 2, and motif 5, which encode about 50 amino acids, and they are related to the SRF-box domain (Ng et al., 2001). Sweet potato is a hexaploid plant with both homologous and heterologous chromosomes, which has a complex genetic background (Yang et al., 2017). MCScanX analysis showed that a large number of genes had collinear relationships within the sweet potato genome, and the MADS-box genes also had a certain homology of evolutionary relationship. Among them, 19 MADS-box-genes had collinear relationships. In particular, reciprocal segmental duplications existed in *IbMADS4*, *IbMADS14*, *IbMADS32*, and *IbMADS56*. *IbMAD51* shared a segmental duplication relationship with *IbMADS4* and *IbMADS56*, while it did not have a segmental duplication relationship with *IbMADS14* and *IbMADS32*. It is presumed that *IbMADS4*, *IbMADS14*, *IbMADS32*, and *IbMADS56* were derived from a single duplication event, while *IbMAD51* was derived from another separate duplication event, possibly a single segmental duplication event.

The MADS-box family is considered to be as a key family of genes responsible for regulating reproductive growth and vegetative organ development, which was initially characterized as homeotic genes in floral organs. Also, the importance of the MADS-box family in the morphogenesis and growth of other organs of plants, especially roots and fruits, has been highlighted (Riechmann et al., 1997; Zhao et al., 2019). Multiple types of *cis*-acting elements have been identified in the promoter region of the MADS-box gene from sweet potato, mainly including light-responsive elements, auxin-responsive elements, MeJA-responsive elements, abscisic acid-responsive elements, and drought-inducible elements. It is indicated that MADS-box genes in sweet potato respond to both hormones and environmental factors, and they are closely related to the normal growth and development of sweet potato. Expression levels of MADS-box genes can reveal the relevant physiological functions, Hasebe et al. (1998) found that most MADS-box genes in ferns are expressed in reproductive and vegetative organs, while most MADS-box genes in seed plants are only expressed in one organism, and only a few are expressed in reproductive and vegetative organisms. Transcriptome sequencing revealed that the majority of MADS-box genes in sweet potato exhibit the expression specialization. Thirty-four genes were specifically expressed in sweet potato flowers and fruits, while the remaining were mainly expressed in vegetative tissues like the root and stem tip. The expression profiles of 10 genes in different tissues of sweet potato were further verified by qRT-PCR. *IbMADS1* was highly expressed in the fibrous root; and *IbMADS79*, *IbMADS18*, *IbMADS19*, and *IbMADS90* were highly expressed in the tuberous root. It has been reported that *IbMADS1* and *IbMADS*79 are associated with the root development of sweet potato. In particular, *IbMADS1* is an important integrator at the initiation of tuberization (Kim et al., 2005; Ku et al., 2008), which is consistent with our finding. *Cis*-acting elements like the light-responsive elements, Zein metabolism-regulation elements, and auxin-responsive elements were found in the promoter region of *IbMADS1*; and light-responsive elements, meristem-express elements, MeJA-responsive elements, and salicylic acid-responsive element were found in the promoter region of *IbMADS79*. *IbMADS1* and *IbMADS*79 may play an important role in the root development process and may participate in the formation of the tuberous root. The homologous genes *IbMADS18* and *IbMADS19* of *FEM11* were highly expressed not only in the fruit but also in the tuberous root. And the homologous gene *IbMADS90* of *AGL62* was highly expressed in the tuberous root and leaf. *FEM111* and *AGL62* are associated with the flower and fruit development in *Arabidopsis* (Kang et al., 2008; Portereiko et al., 2006; Steffen et al., 2008). *IbMADS18*, *IbMADS19*, and *IbMADS90* are involved in the tuberous root development of sweet potato and the fruit development to some extent. In addition, *IbMADS31* and *IbMADS83* are also specifically expressed in the fruit. *AGL61*, a homolog of *IbMADS83*, functions as a TF that controls the expression of downstream genes during central cell development and is involved in the seed endosperm development (Steffen et al., 2008). And *AGL17*, the homolog of *IbMADS31*, is a significant downstream target of CURLY LEAF in floral transition control (Shu et al., 2020). We found that *cis*-acting elements like the MeJA-responsive elements, auxin-responsive elements, abscisic acid-responsive elements, and gibberellin-responsive elements in the promoter region of *IbMADS31* and *IbMADS83* provided indirect evidences for the involvement of *IbMADS31* and *IbMADS83* in the flower and fruit development of sweet potato. *IbMADS15*, *IbMADS17*, *IbMADS19*, *IbMADS20*, *IbMADS79*, and *IbMADS90* were highly expressed in the leaf or stem, which were all identified as type I genes. In addition, a large number of type I genes were detected mainly expressed in the stem tip by RNA-seq. It is suggested that type I genes were involved in the nutritional growth process of the aerial parts of sweet potato, to some extent.

Overall, the sweet potato MADS-box genes are involved in the growth and development of reproductive organs like the flower and fruit of sweet potato. They were vital in the process of nutritional growth and development such as the root, stem, and leaf of sweet potato.

## 5 Conclusion

In this study, the 95 MADS-box family genes were identified from the sweet potato genome by Hmmer, SMART, and other tools, and they were divided into type I and type II subgroups according to the gene structure. The phylogenetic relationship of MADS-box genes between sweet potato and *Arabidopsis* was analyzed based on the NJ phylogenetic tree. The type I genes of sweet potato were further divided into three subfamilies, M*α*, M*β*, and M*γ*, whereas the type II genes were divided into 12 subfamilies.

The distribution of the sweet potato MADS-box genes on chromosomes was analyzed by chromosomal localization, and the analysis of the motif identified the MADS-box conserved domain motifs. Collinearity between and within chromosomes of sweet potato was analyzed using MCScanX to identify 19 MADS-box genes with segmental duplications or tandem duplications. Analysis of the MADS-box promoter region of sweet potato revealed that the MADS-box genes contain light-responsive elements, auxin-responsive elements, MeJA-responsive elements, abscisic acid-responsive elements, drought induction elements, and other *cis*-acting elements that respond to multiple environmental changes and coordinate growth and development. Furthermore, we analyzed the expression patterns of MADS-box genes in various tissues of sweet potato by transcriptome sequencing and qRT-PCR, and we found that the sweet potato MADS-box genes are involved in the growth and development of reproductive organs such as the flower and fruit, but also the growth and development of nutritional organs such as the root, stem, and leaf of sweet potato.

## Data Availability

The datasets presented in this study can be found in online repositories. The names of the repository/repositories and accession number(s) can be found in the article/Supplementary Material.

## References

[B1] Bai G. Yang D. H. Cao P. Yao H. Zhang Y. Chen X. (2019). Genome-Wide Identification, Gene Structure and Expression Analysis of the MADS-Box Gene Family Indicate Their Function in the Development of Tobacco (Nicotiana Tabacum l). Int. J. Mol. Sci. 20 (20), 5043. 10.3390/ijms20205043 PMC6829366 31614589

[B2] Bailey T. L. Boden M. Buske F. A. Frith M. Grant C. E. Clementi L. (2009). MEME SUITE: Tools for Motif Discovery and Searching. Nucleic Acids Res. 37 (2), W202–W208. 10.1093/nar/gkp335 19458158 PMC2703892

[B3] Burgeff C. Liljegren S. J. Tapia-López R. Yanofsky M. F. Alvarez-Buylla E. R. (2002). MADS-Box Gene Expression in Lateral Primordia, Meristems and Differentiated Tissues of *Arabidopsis thaliana* Roots. Planta 214 (3), 365–372. 10.1007/s004250100637 11855641

[B4] Chen C. Chen H. Zhang Y. Thomas H. R. Frank M. H. He Y. (2020). TBtools: An Integrative Toolkit Developed for Interactive Analyses of Big Biological Data. Mol. Plant 13 (8), 1194–1202. 10.1016/j.molp.2020.06.009 32585190

[B5] De Bodt S. Raes J. Florquin K. Rombauts S. Rouz P. Theien G. (2003). Genomewide Structural Annotation and Evolutionary Analysis of the Type I MADS-Box Genes in Plants. J. Mol. Evol. 56 (5), 573–586. 10.1007/s00239-002-2426-x 12698294

[B6] Dong T. Song W. Tan C. Zhou Z. Yu J. Han R. (2018). Molecular Characterization of Nine Sweet Potato (Ipomoea Batatas Lam.) MADS-Box Transcription Factors during Storage Root Development and Following Abiotic Stress. Plant Breed 137 (5), 790–804. 10.1111/pbr.12613

[B7] Duan W. Song X. Liu T. Huang Z. Ren J. Hou X. (2015). Genome-wide Analysis of the MADS-Box Gene Family in Brassica Rapa (Chinese Cabbage). Mol. Genet. Genomics. 290 (1), 239–255. 10.1007/s00438-014-0912-7 25216934

[B8] Fan H.-Y. Hu Y. Tudor M. Ma H. (1997). Specific Interactions between the K Domains of AG and AGLs, Members of the MADS Domain Family of DNA Binding Proteins. Plant J. 12 (5), 999–1010. 10.1046/j.1365-313X.1997.12050999.x 9418042

[B9] Florea L. Song L. Salzberg S. L. (2013). Thousands of Exon Skipping Events Differentiate Among Splicing Patterns in Sixteen Human Tissues. F1000Res 2, 188. 10.12688/f1000research.2-188.v2 24555089 PMC3892928

[B10] Ghorbani Marghashi M. Bagheri H. Gholami M. (2020). Genome-Wide Study of Flowering-Related MADS-Box Genes Family in Cardamine Hirsuta. 3 Biotech. 10 (12), 518. 10.1007/s13205-020-02521-w PMC7652958 33194522

[B11] Hasebe M. Wen C.-K. Kato M. Banks J. A. (1998). Characterization of MADS Homeotic Genes in the Fern Ceratopteris Richardii. Proc. Natl. Acad. Sci. 95 (11), 6222–6227. 10.1073/pnas.95.11.6222 9600946 PMC27636

[B12] Ito K. Murphy D. (2013). Application of Ggplot2 to Pharmacometric Graphics. CPT: Pharmacometrics Syst. Pharmacol. 2, 79. 10.1038/psp.2013.56 PMC3817376 24132163

[B13] Jin J. Tian F. Yang D.-C. Meng Y.-Q. Kong L. Luo J. (2017). PlantTFDB 4.0: Toward a Central Hub for Transcription Factors and Regulatory Interactions in Plants. Nucleic Acids Res. 45 (D1), D1040–D1045. 10.1093/nar/gkw982 27924042 PMC5210657

[B14] Kang I.-H. Steffen J. G. Portereiko M. F. Lloyd A. Drews G. N. (2008). The AGL62 MADS Domain Protein Regulates Cellularization during Endosperm Development inArabidopsis. Plant Cell 20 (3), 635–647. 10.1105/tpc.107.055137 18334668 PMC2329934

[B15] Kaufmann K. Melzer R. Theissen G. (2005). MIKC-Type MADS-Domain Proteins: Structural Modularity, Protein Interactions and Network Evolution in Land Plants. Gene 347 (2), 183–198. 10.1016/j.gene.2004.12.014 15777618

[B16] Kim S.-H. Hamada T. Otani M. Shimada T. (2005). Isolation and Characterization of MADS Box Genes Possibly Related to Root Development in Sweetpotato(Ipomoea Batatas L. Lam). J. Plant Biol. 48 (4), 387–393. 10.1007/BF03030580

[B17] Kim S.-H. Mizuno K. Fujimura T. (2002). Isolation of MADS-Box Genes from Sweet Potato (Ipomoea Batatas (L.) Lam.) Expressed Specifically in Vegetative Tissues. Plant Cel Physiol 43 (3), 314–322. 10.1093/pcp/pcf043 11917086

[B18] Krejci A. Hupp T. R. Lexa M. Vojtesek B. Muller P. (2016). Hammock: A Hidden Markov Model-Based Peptide Clustering Algorithm to Identify Protein-Interaction Consensus Motifs in Large Datasets. Bioinformatics 32 (1), btv522–16. 10.1093/bioinformatics/btv522 PMC4681989 26342231

[B19] Krzywinski M. Schein J. Birol I. Connors J. Gascoyne R. Horsman D. (2009). Circos: An Information Aesthetic for Comparative Genomics. Genome Res. 19 (9), 1639–1645. 10.1101/gr.092759.109 19541911 PMC2752132

[B20] Ku A. T. Huang Y.-S. Wang Y.-S. Ma D. Yeh K.-W. (2008). IbMADS1 (Ipomoea Batatas MADS-Box 1 Gene) Is Involved in Tuberous Root Initiation in Sweet Potato (Ipomoea Batatas). Ann. Bot. 102 (1), 57–67. 10.1093/aob/mcn067 18463111 PMC2712425

[B21] Kumar S. Stecher G. Li M. Knyaz C. Tamura K. (2018). MEGA X: Molecular Evolutionary Genetics Analysis across Computing Platforms. Mol. Biol. Evol. 35 (6), 1547–1549. 10.1093/molbev/msy096 29722887 PMC5967553

[B22] Lamesch P. Berardini T. Z. Li D. Swarbreck D. Wilks C. Sasidharan R. (2012). The Arabidopsis Information Resource (TAIR): Improved Gene Annotation and New Tools. Nucleic Acids Res. 40 (Database issue), D1202–D1210. 10.1093/nar/gkr1090 22140109 PMC3245047

[B23] Lescot M. Dehais P. Thijs G. Marchal K. Moreau Y. Van de Peer Y. (2002). PlantCARE, a Database of Plant Cis-Acting Regulatory Elements and a portal to Tools for In Silico Analysis of Promoter Sequences. Nucleic Acids Res. 30 (1), 325–327. 10.1093/nar/30.1.325 11752327 PMC99092

[B24] Livak K. J. Schmittgen T. D. (2001). Analysis of Relative Gene Expression Data Using Real-Time Quantitative PCR and the 2−ΔΔCT Method. Methods 25 (4), 402–408. 10.1006/meth.2001.1262 11846609

[B25] Michaels S. D. Amasino R. M. (1999). FLOWERING LOCUS C Encodes a Novel MADS Domain Protein that Acts as a Repressor of Flowering. Plant Cell 11 (5), 949–956. 10.1105/tpc.11.5.949 10330478 PMC144226

[B26] Michaels S. D. He Y. Scortecci K. C. Amasino R. M. (2003). Attenuation of FLOWERING LOCUS C Activity as a Mechanism for the Evolution of Summer-Annual Flowering Behavior in Arabidopsis. Proc. Natl. Acad. Sci. 100 (17), 10102–10107. 10.1073/pnas.1531467100 12904584 PMC187779

[B27] Nam J. Kaufmann K. Theissen G. Nei M. (2005). A Simple Method for Predicting the Functional Differentiation of Duplicate Genes and its Application to MIKC-Type MADS-Box Genes. Nucleic Acids Res. 33 (2), e12. 10.1093/nar/gni003 15659573 PMC548370

[B28] Nesi N. Debeaujon I. Jond C. Stewart A. J. Jenkins G. I. Caboche M. (2002). The Transparent Testa16 Locus Encodes the Arabidopsis Bsister Mads Domain Protein and Is Required for Proper Development and Pigmentation of the Seed Coat. Plant Cell 14 (10), 2463–2479. 10.1105/tpc.004127 12368498 PMC151229

[B29] Ng M. Yanofsky M. F. (2001). Function and Evolution of the Plant MADS-Box Gene Family. Nat. Rev. Genet. 2 (3), 186–195. 10.1038/35056041 11256070

[B30] Norman C. Runswick M. Pollock R. Treisman R. (1988). Isolation and Properties of cDNA Clones Encoding SRF, a Transcription Factor that Binds to the C-Fos Serum Response Element. Cell 55 (6), 989–1003. 10.1016/0092-8674(88)90244-9 3203386

[B31] Par̆enicová L. de Folter S. Kieffer M. Horner D. S. Favalli C. Busscher J. (2003). Molecular and Phylogenetic Analyses of the Complete MADS-Box Transcription Factor Family in Arabidopsis. Plant Cell 15 (7), 1538–1551. 10.1105/tpc.011544 12837945 PMC165399

[B32] Passmore S. Maine G. T. Elble R. Christ C. Tye B.-K. (1988). *Saccharomyces cerevisiae* Protein Involved in Plasmid Maintenance Is Necessary for Mating of MATα Cells. J. Mol. Biol. 204 (3), 593–606. 10.1016/0022-2836(88)90358-0 3066908

[B33] Portereiko M. F. Lloyd A. Steffen J. G. Punwani J. A. Otsuga D. Drews G. N. (2006). AGL80Is Required for Central Cell and Endosperm Development inArabidopsis. Plant Cell 18 (8), 1862–1872. 10.1105/tpc.106.040824 16798889 PMC1533969

[B34] Ray S. Agarwal P. Arora R. Kapoor S. Tyagi A. K. (2007). Expression Analysis of Calcium-Dependent Protein Kinase Gene Family during Reproductive Development and Abiotic Stress Conditions in rice (Oryza Sativa L. Ssp. Indica). Mol. Genet. Genomics. 278 (5), 493–505. 10.1007/s00438-007-0267-4 17636330

[B35] Riechmann J. L. Meyerowitz E. M. (1997). MADS Domain Proteins in Plant Development. Biol. Chem. 378 (10), 1079–1101. 10.1515/bchm.1997.378.10.1079 9372178

[B36] Saitou N. Nei M. (1987). The Neighbor-Joining Method: A New Method for Reconstructing Phylogenetic Trees. Mol. Biol. Evol. 4 (4), 406–425. 10.1093/oxfordjournals.molbev.a040454 3447015

[B37] Sheng X.-G. Zhao Z.-Q. Wang J.-S. Yu H.-F. Shen Y.-S. Zeng X.-Y. (2019). Genome Wide Analysis of MADS-Box Gene Family in *Brassica oleracea* Reveals Conservation and Variation in Flower Development. BMC Plant Biol. 19 (1), 106. 10.1186/s12870-019-1717-y 30890145 PMC6425688

[B38] Shu J. Chen C. Kohalmi S. E. Cui Y. (2020). Evidence that AGL17 Is a Significant Downstream Target of CLF in floral Transition Control. Plant Signaling Behav. 15 (7), 1766851. 10.1080/15592324.2020.1766851 PMC8570702 32408840

[B39] Singh V. K. Mangalam A. K. Dwivedi S. Naik S. (1998). Primer Premier: Program for Design of Degenerate Primers from a Protein Sequence. Biotechniques 24 (2), 318–319. 10.2144/98242pf02 9494736

[B40] Sommer H. Beltrán J. P. Huijser P. Pape H. Lönnig W. E. Saedler H. (1990). Deficiens, a Homeotic Gene Involved in the Control of Flower Morphogenesis in Antirrhinum Majus: The Protein Shows Homology to Transcription Factors. EMBO J. 9 (3), 605–613. 10.1002/j.1460-2075.1990.tb08152.x 1968830 PMC551713

[B41] Steffen J. G. Kang I.-H. Portereiko M. F. Lloyd A. Drews G. N. (2008). AGL61 Interacts with AGL80 and Is Required for Central Cell Development in Arabidopsis. Plant Physiol. 148 (1), 259–268. 10.1104/pp.108.119404 18599653 PMC2528130

[B42] Sumiya N. (2021). Cis-Acting Elements Involved in the G2/M-Phase-Specific Transcription of the Cyclin B Gene in the Unicellular Alga Cyanidioschyzon Merolae. J. Plant Res. 134 (6), 1301–1310. 10.1007/s10265-021-01334-z 34338916

[B43] Sun C.-H. Wang J.-H. Gu K.-D. Zhang P. Zhang X.-Y. Zheng C.-S. (2021). New Insights into the Role of MADS-Box Transcription Factor Gene CmANR1 on Root and Shoot Development in chrysanthemum (Chrysanthemum Morifolium). BMC Plant Biol. 21 (1), 79. 10.1186/s12870-021-02860-7 33549046 PMC7866475

[B44] Tang Y. Wang J. Bao X. Wu Q. Yang T. Li H. (2020). Genome-Wide Analysis of Jatropha Curcas MADS-Box Gene Family and Functional Characterization of the JcMADS40 Gene in Transgenic rice. BMC Genomics 21 (1), 325. 10.1186/s12864-020-6741-7 32345214 PMC7187513

[B45] Theissen G. (2001). Development of floral Organ Identity: Stories from the MADS House. Curr. Opin. Plant Biol. 4 (1), 75–85. 10.1016/s1369-5266(00)00139-4 11163172

[B46] Voorrips R. E. (2002). MapChart: Software for the Graphical Presentation of Linkage Maps and QTLs. J. Hered. 93 (1), 77–78. 10.1093/jhered/93.1.77 12011185

[B47] Wang S. Nie S. Zhu F. (2016). Chemical Constituents and Health Effects of Sweet Potato. Food Res. Int. 89 (Pt 1), 90–116. 10.1016/j.foodres.2016.08.032 28460992

[B48] Wang Y. Tang H. Debarry J. D. Tan X. Li J. Wang X. (2012). MCScanX: A Toolkit for Detection and Evolutionary Analysis of Gene Synteny and Collinearity. Nucleic Acids Res. 40 (7), e49. 10.1093/nar/gkr1293 22217600 PMC3326336

[B49] Wang Y. Zhang J. Hu Z. Guo X. Tian S. Chen G. (2019). Genome-Wide Analysis of the MADS-Box Transcription Factor Family in Solanum Lycopersicum. Int. J. Mol. Sci. 20 (12), 2961. 10.3390/ijms20122961 PMC6627509 31216621

[B50] Won S. Y. Jung J.-A. Kim J. S. (2021). Genome-Wide Analysis of the MADS-Box Gene Family in Chrysanthemum. Comput. Biol. Chem. 90, 107424. 10.1016/j.compbiolchem.2020.107424 33340990

[B51] Wu Z. Raven P. H. Hong D. (1998). Flora of china. Beijing: Science Press.

[B52] Yang J. Moeinzadeh M.-H. Kuhl H. Helmuth J. Xiao P. Haas S. (2017). Haplotype-Resolved Sweet Potato Genome Traces Back its Hexaploidization History. Nat. Plants 3 (9), 696–703. 10.1038/s41477-017-0002-z 28827752

[B53] Yanofsky M. F. Ma H. Bowman J. L. Drews G. N. Feldmann K. A. Meyerowitz E. M. (1990). The Protein Encoded by the Arabidopsis Homeotic Gene Agamous Resembles Transcription Factors. Nature 346 (6279), 35–39. 10.1038/346035a0 1973265

[B54] Zhang S. Guo Y. Zhang Y. Guo J. Li K. Fu W. (2021). Genome-wide Identification, Characterization and Expression Profiles of the CCD Gene Family in Gossypium Species. 3 Biotech. 11 (5), 249. 10.1007/s13205-021-02805-9 PMC8088422 33968592

[B55] Zhao H.-B. Jia H.-M. Wang Y. Wang G.-Y. Zhou C.-C. Jia H.-J. (2019). Genome-Wide Identification and Analysis of the MADS-Box Gene Family and its Potential Role in Fruit Development and Ripening in Red Bayberry (Morella Rubra). Gene 717, 144045. 10.1016/j.gene.2019.144045 31425741

[B56] Zhu P. Dong T. Xu T. Kang H. (2020). Identification, Characterisation and Expression Analysis of MADS-Box Genes in Sweetpotato Wild Relative Ipomoea Trifida. Acta Physiol. Plant 42 (11), 163. 10.1007/s11738-020-03153-6

