# Supplementary material for: Genome-Wide Identification and Expression Analysis of the MADS-Box Gene Family in Sweet Potato [Ipomoea batatas (L.) Lam]
Source: Front Genet. 2021 Nov 17;12:750137. doi: 10.3389/fgene.2021.750137 (PMC8636027; doi:10.3389/fgene.2021.750137)
Supplement: Supplementary file 1 [file Table1.docx]

Supplementary Material

**Supplementary Table S1.** qPCR primers and their sequences used in this study.

| **Primer name** | **Forward Sequence (5’ to 3’)** | **Reverse Sequence (5’ to 3’)** |
| --- | --- | --- |
| *β-actin* | TCCAGAAGAGCACCCGGTAC | GTCTGTCAGGTCACGTCCAG |
| *IbMADS1* | GGAAATGAGTTTGAGCGGCA | TGGCCTTGGGATTCTTCACT |
| *IbMADS15* | TGAGTGCGGTGAAGGAATCT | AGCATACCTCGTCCGCATTA |
| *IbMADS17* | AACCGAGGTGATGTTCCAGT | AAATCTATCCCCGCCTCCTG |
| *IbMADS18* | TCATCACCAACGACTCAGCT | TCTTGCTCTGCTCCATCTCC |
| *IbMADS19* | AACCGAGGTGATGTTCCAGT | TCATCCAATCCGCAAACCAC |
| *IbMADS20* | AACCGAGGTGATGTTCCAGT | AAATCTATCCCCGCCTCCTG |
| *IbMADS31* | GTTGAAGAAGGCCAAGGAGC | GCCATGAGTTCTGATGCTGG |
| *IbMADS79* | CAGCCGTTCTACATCCTTGC | ACCTGCAATGCTCTCTCCTT |
| *IbMADS83* | AGACGAAGACTCACCTGCAA | GAGAGGAACCGGTCGAGAAT |
| *IbMADS90* | ACAAGGTTTTCTCGTTCGGC | CTCCCTGTTGAGCTCCTGAA |
