# Supplementary material for: Genome-Wide Identification and Expression Analysis of the MADS-Box Gene Family in Sweet Potato [Ipomoea batatas (L.) Lam]
Source: Front Genet. 2021 Nov 17;12:750137. doi: 10.3389/fgene.2021.750137 (PMC8636027; doi:10.3389/fgene.2021.750137)
Supplement: Supplementary file 2 [file Table2.docx]

Supplementary Material

**Supplementary Table S1.** MADS-box genes identified in sweet potato

| **Gene name** | **Locus ID** | **ORF lenth** | **Chro** | **Extrons** | **Chromosome location** | | **Pfam** |
| --- | --- | --- | --- | --- | --- | --- | --- |
| *IbMADS1* | g38619 | 216 | LG10 | 6 | 3082100 | 3088682 | MADS-domain + K-box |
| *IbMADS2* | g1670 | 61 | LG1 | 1 | 10557784 | 10558283 | MADS-domain |
| *IbMADS3* | g4395 | 199 | LG2 | 1 | 1504468 | 1505234 | MADS-domain |
| *IbMADS4* | g60235 | 227 | LG15 | 7 | 2200857 | 2204768 | MADS-domain + K-box |
| *IbMADS5* | g5812 | 64 | LG2 | 1 | 10857424 | 10857968 | MADS-domain |
| *IbMADS6* | g8896 | 72 | LG2 | 2 | 34399534 | 34401119 | MADS-domain |
| *IbMADS7* | g9269 | 888 | LG2 | 17 | 36755916 | 36765626 | MADS-domain + K-box |
| *IbMADS8* | g13049 | 75 | LG4 | 2 | 1940438 | 1941933 | MADS-domain |
| *IbMADS9* | g13050 | 240 | LG4 | 8 | 1947630 | 1952173 | MADS-domain + K-box |
| *IbMADS10* | g13580 | 70 | LG4 | 2 | 5337438 | 5338636 | MADS-domain |
| *IbMADS11* | g14156 | 314 | LG4 | 4 | 9870185 | 9872832 | MADS-domain |
| *IbMADS12* | g15818 | 61 | LG4 | 1 | 22978937 | 22979878 | MADS-domain |
| *IbMADS13* | g16277 | 244 | LG4 | 1 | 26828409 | 26829470 | MADS-domain |
| *IbMADS14* | g16795 | 296 | LG5 | 10 | 1212374 | 1216043 | MADS-domain + K-box |
| *IbMADS15* | g17656 | 337 | LG5 | 4 | 7105366 | 7106740 | MADS-domain |
| *IbMADS16* | g17661 | 303 | LG5 | 1 | 7130450 | 7131640 | MADS-domain |
| *IbMADS17* | g20955 | 239 | LG6 | 1 | 502507 | 503419 | MADS-domain |
| *IbMADS18* | g20956 | 228 | LG6 | 1 | 505507 | 506571 | MADS-domain |
| *IbMADS19* | g22981 | 231 | LG6 | 1 | 15794121 | 15797499 | MADS-domain |
| *IbMADS20* | g22984 | 235 | LG6 | 1 | 15805409 | 15806309 | MADS-domain |
| *IbMADS21* | g23435 | 204 | LG6 | 2 | 19027253 | 19028189 | MADS-domain |
| *IbMADS22* | g23438 | 251 | LG6 | 1 | 19034806 | 19035739 | MADS-domain |
| *IbMADS23* | g23441 | 231 | LG6 | 1 | 19056330 | 19057259 | MADS-domain |
| *IbMADS24* | g25324 | 73 | LG7 | 2 | 353195 | 354445 | MADS-domain |
| *IbMADS25* | g25331 | 165 | LG7 | 4 | 404367 | 407036 | MADS-domain |
| *IbMADS26* | g26088 | 142 | LG7 | 1 | 5400030 | 5400569 | MADS-domain |
| *IbMADS27* | g26120 | 191 | LG7 | 1 | 5614540 | 5615357 | MADS-domain |
| *IbMADS28* | g26122 | 191 | LG7 | 1 | 5623929 | 5626997 | MADS-domain |
| *IbMADS29* | g26124 | 147 | LG7 | 1 | 5633842 | 5634787 | MADS-domain |
| *IbMADS30* | g27471 | 193 | LG7 | 1 | 16552648 | 16553564 | MADS-domain |
| *IbMADS31* | g29145 | 273 | LG7 | 9 | 28348685 | 28353827 | MADS-domain + K-box |
| *IbMADS32* | g29830 | 227 | LG7 | 8 | 33107161 | 33109073 | MADS-domain + K-box |
| *IbMADS33* | g30020 | 241 | LG7 | 8 | 34286345 | 34289838 | MADS-domain + K-box |
| *IbMADS34* | g30372 | 328 | LG8 | 10 | 714718 | 719046 | MADS-domain + K-box |
| *IbMADS35* | g30373 | 299 | LG8 | 10 | 719525 | 725203 | MADS-domain + K-box |
| *IbMADS36* | g31969 | 75 | LG8 | 1 | 11249825 | 11250903 | MADS-domain |
| *IbMADS37* | g31975 | 71 | LG8 | 1 | 11282662 | 11283763 | MADS-domain |
| *IbMADS38* | g31995 | 254 | LG8 | 8 | 11439103 | 11443426 | MADS-domain + K-box |
| *IbMADS39* | g33383 | 208 | LG8 | 2 | 22254213 | 22255559 | MADS-domain |
| *IbMADS40* | g33384 | 278 | LG8 | 3 | 22255853 | 22258889 | MADS-domain |
| *IbMADS41* | g33385 | 267 | LG8 | 4 | 22259181 | 22262389 | MADS-domain |
| *IbMADS42* | g33422 | 208 | LG8 | 2 | 22609658 | 22611041 | MADS-domain |
| *IbMADS43* | g33443 | 203 | LG8 | 1 | 22745260 | 22746347 | MADS-domain |
| *IbMADS44* | g33446 | 206 | LG8 | 1 | 22763290 | 22764440 | MADS-domain |
| *IbMADS45* | g34250 | 134 | LG9 | 4 | 1581991 | 1585012 | MADS-domain |
| *IbMADS46* | g34704 | 77 | LG9 | 1 | 4421434 | 4423107 | MADS-domain |
| *IbMADS47* | g35098 | 149 | LG9 | 2 | 7373609 | 7374250 | MADS-domain |
| *IbMADS48* | g35561 | 259 | LG9 | 2 | 10872930 | 10875769 | MADS-domain |
| *IbMADS49* | g38545 | 232 | LG10 | 2 | 2569706 | 2571196 | MADS-domain |
| *IbMADS50* | g38862 | 232 | LG10 | 7 | 4766349 | 4769830 | MADS-domain + K-box |
| *IbMADS51* | g38982 | 225 | LG10 | 7 | 5727528 | 5733141 | MADS-domain + K-box |
| *IbMADS52* | g39426 | 61 | LG10 | 1 | 9162531 | 9167882 | MADS-domain |
| *IbMADS53* | g40925 | 200 | LG10 | 1 | 20345450 | 20346444 | MADS-domain |
| *IbMADS54* | g41032 | 213 | LG10 | 2 | 21289569 | 21290637 | MADS-domain |
| *IbMADS55* | g42006 | 118 | LG11 | 2 | 4048057 | 4049406 | MADS-domain |
| *IbMADS56* | g42031 | 221 | LG11 | 6 | 4206130 | 4210068 | MADS-domain + K-box |
| *IbMADS57* | g42033 | 79 | LG11 | 1 | 4218969 | 4219938 | MADS-domain |
| *IbMADS58* | g42247 | 203 | LG11 | 7 | 5631383 | 5634288 | MADS-domain + K-box |
| *IbMADS59* | g42393 | 106 | LG11 | 3 | 6818862 | 6822500 | MADS-domain |
| *IbMADS60* | g42798 | 802 | LG11 | 17 | 9654445 | 9661263 | MADS-domain |
| *IbMADS61* | g43053 | 220 | LG11 | 7 | 11385792 | 11389139 | MADS-domain + K-box |
| *IbMADS62* | g43195 | 231 | LG11 | 7 | 12484457 | 12486492 | MADS-domain + K-box |
| *IbMADS63* | g46705 | 201 | LG11 | 7 | 39175343 | 39182481 | MADS-domain + K-box |
| *IbMADS64* | g46715 | 359 | LG11 | 10 | 39234183 | 39243211 | MADS-domain + K-box |
| *IbMADS65* | g46738 | 231 | LG11 | 1 | 39389083 | 39389938 | MADS-domain |
| *IbMADS66* | g49748 | 234 | LG12 | 1 | 21303859 | 21304817 | MADS-domain |
| *IbMADS67* | g50540 | 61 | LG12 | 1 | 27249192 | 27250980 | MADS-domain |
| *IbMADS68* | g52965 | 297 | LG13 | 1 | 13146724 | 13148531 | MADS-domain |
| *IbMADS69* | g53008 | 305 | LG13 | 1 | 13390265 | 13392055 | MADS-domain |
| *IbMADS70* | g53189 | 70 | LG13 | 2 | 14818284 | 14819343 | MADS-domain |
| *IbMADS71* | g53866 | 186 | LG13 | 1 | 20091405 | 20092323 | MADS-domain |
| *IbMADS72* | g54366 | 139 | LG13 | 1 | 23434030 | 23434776 | MADS-domain |
| *IbMADS73* | g54651 | 326 | LG13 | 8 | 25155054 | 25161201 | MADS-domain + K-box |
| *IbMADS74* | g57201 | 153 | LG14 | 1 | 13105151 | 13105915 | MADS-domain |
| *IbMADS75* | g57754 | 374 | LG14 | 5 | 17245174 | 17247598 | MADS-domain |
| *IbMADS76* | g57755 | 181 | LG14 | 1 | 17249644 | 17250501 | MADS-domain |
| *IbMADS77* | g57793 | 295 | LG14 | 9 | 17550753 | 17554541 | MADS-domain + K-box |
| *IbMADS78* | g57866 | 368 | LG14 | 1 | 18118573 | 18119897 | MADS-domain |
| *IbMADS79* | g30018 | 271 | LG7 | 9 | 34271456 | 34276098 | MADS-domain |
| *IbMADS80* | g57868 | 1108 | LG14 | 9 | 18131742 | 18136497 | MADS-domain |
| *IbMADS81* | g59412 | 426 | LG14 | 1 | 28526755 | 28534723 | MADS-domain |
| *IbMADS82* | g59555 | 131 | LG14 | 4 | 29290213 | 29295712 | MADS-domain |
| *IbMADS83* | g59580 | 167 | LG14 | 1 | 29402992 | 29403726 | MADS-domain |
| *IbMADS84* | g60020 | 227 | LG15 | 8 | 892308 | 895598 | MADS-domain + K-box |
| *IbMADS85* | g60104 | 99 | LG15 | 2 | 1463202 | 1465820 | MADS-domain |
| *IbMADS86* | g60370 | 333 | LG15 | 11 | 3104037 | 3106727 | MADS-domain |
| *IbMADS87* | g61427 | 298 | LG15 | 2 | 10413181 | 10415305 | MADS-domain |
| *IbMADS88* | g61428 | 167 | LG15 | 1 | 10417461 | 10418264 | MADS-domain |
| *IbMADS89* | g61430 | 211 | LG15 | 4 | 10434772 | 10437046 | MADS-domain |
| *IbMADS90* | g61431 | 270 | LG15 | 1 | 10438165 | 10439276 | MADS-domain |
| *IbMADS91* | g61434 | 205 | LG15 | 2 | 10459972 | 10460976 | MADS-domain |
| *IbMADS92* | g61441 | 242 | LG15 | 1 | 10492165 | 10493106 | MADS-domain |
| *IbMADS93* | g61442 | 317 | LG15 | 2 | 10494702 | 10496626 | MADS-domain |
| *IbMADS94* | g61443 | 264 | LG15 | 2 | 10500893 | 10502066 | MADS-domain |
| *IbMADS95* | g61444 | 323 | LG15 | 7 | 10505372 | 10510752 | MADS-domain |
